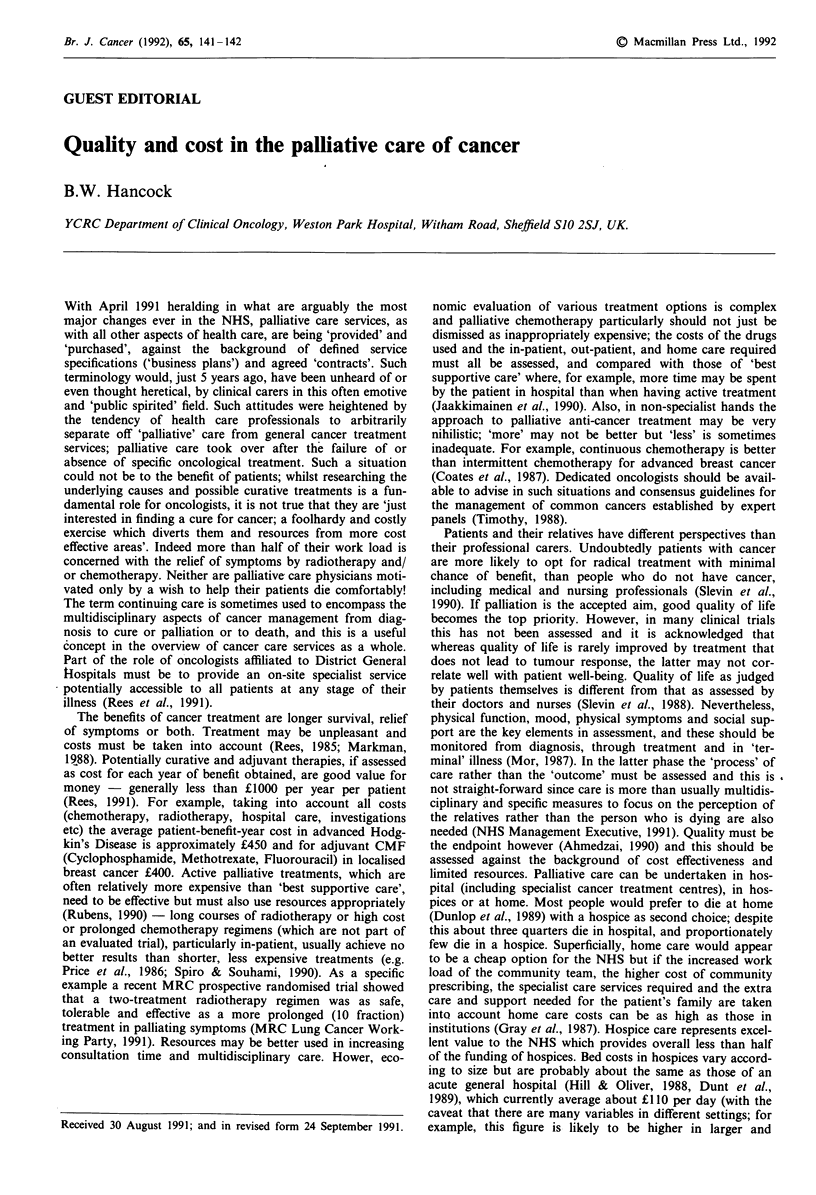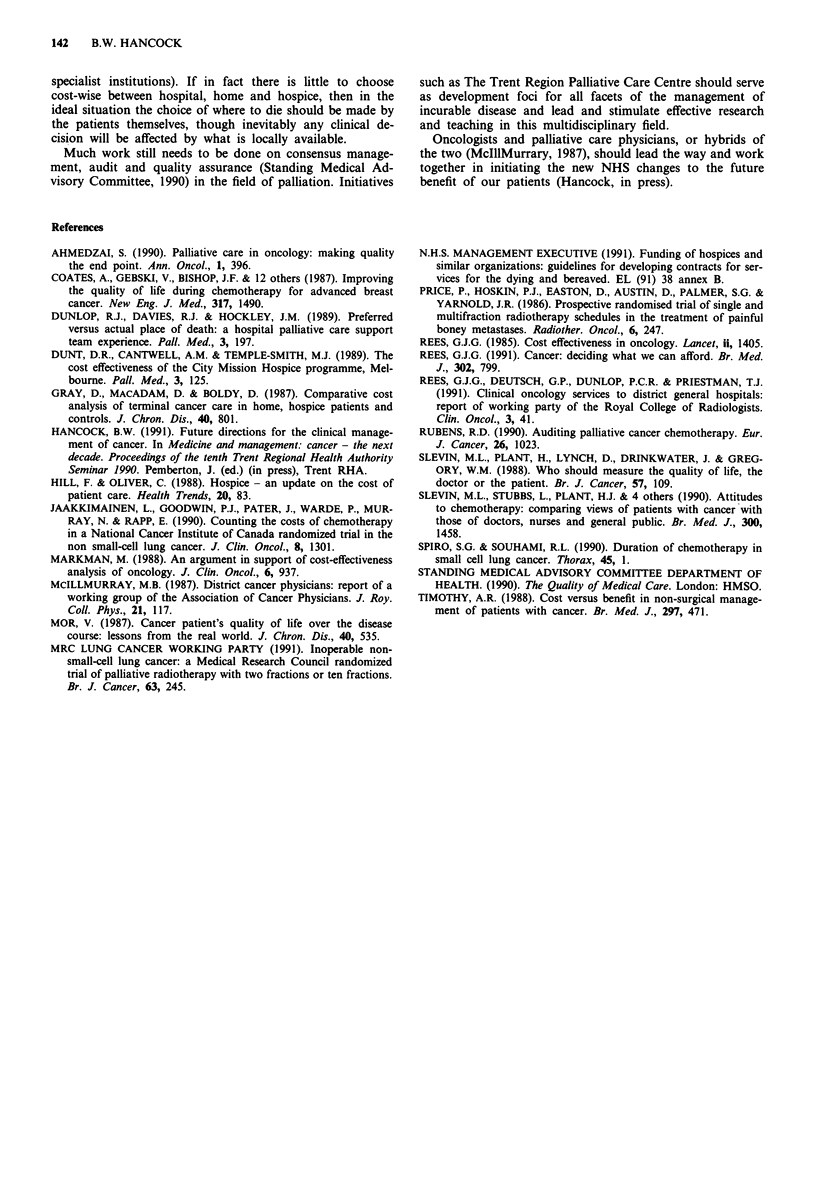# Quality and cost in the palliative care of cancer.

**DOI:** 10.1038/bjc.1992.29

**Published:** 1992-02

**Authors:** B. W. Hancock


Br. J. Cancer (1992), 65, 141–142 © Macmillan Press Ltd., 1992

GUEST EDITORIAL

### Quality and cost in the palliative care of cancer

B.W. Hancock

*YCRC Department of Clinical Oncology, Weston Park Hospital, Witham Road, Sheffield S10 2SJ, UK.*

With April 1991 heralding in what are arguably the most major changes ever in the NHS, palliative care services, as with all other aspects of health care, are being 'provided' and 'purchased', against the background of defined service specifications ('business plans') and agreed 'contracts'. Such terminology would, just 5 years ago, have been unheard of or even thought heretical, by clinical carers in this often emotive and 'public spirited' field. Such attitudes were heightened by the tendency of health care professionals to arbitrarily separate off 'palliative' care from general cancer treatment services; palliative care took over after the failure of or absence of specific oncological treatment. Such a situation could not be to the benefit of patients; whilst researching the underlying causes and possible curative treatments is a fundamental role for oncologists, it is not true that they are 'just interested in finding a cure for cancer; a foolhardy and costly exercise which diverts them and resources from more cost effective areas'. Indeed more than half of their work load is concerned with the relief of symptoms by radiotherapy and/or chemotherapy. Neither are palliative care physicians motivated only by a wish to help their patients die comfortably! The term continuing care is sometimes used to encompass the multidisciplinary aspects of cancer management from diagnosis to cure or palliation or to death, and this is a useful concept in the overview of cancer care services as a whole. Part of the role of oncologists affiliated to District General Hospitals must be to provide an on-site specialist service potentially accessible to all patients at any stage of their illness (Rees *et al.*, 1991).

The benefits of cancer treatment are longer survival, relief of symptoms or both. Treatment may be unpleasant and costs must be taken into account (Rees, 1985; Markman, 1988). Potentially curative and adjuvant therapies, if assessed as cost for each year of benefit obtained, are good value for money — generally less than £1000 per year per patient (Rees, 1991). For example, taking into account all costs (chemotherapy, radiotherapy, hospital care, investigations etc) the average patient-benefit-year cost in advanced Hodgkin's Disease is approximately £450 and for adjuvant CMF (Cyclophosphamide, Methotrexate, Fluorouracil) in localised breast cancer £400. Active palliative treatments, which are often relatively more expensive than 'best supportive care', need to be effective but must also use resources appropriately (Rubens, 1990) — long courses of radiotherapy or high cost or prolonged chemotherapy regimens (which are not part of an evaluated trial), particularly in-patient, usually achieve no better results than shorter, less expensive treatments (e.g. Price *et al.*, 1986; Spiro & Souhami, 1990). As a specific example a recent MRC prospective randomised trial showed that a two-treatment radiotherapy regimen was as safe, tolerable and effective as a more prolonged (10 fraction) treatment in palliating symptoms (MRC Lung Cancer Working Party, 1991). Resources may be better used in increasing consultation time and multidisciplinary care. Hower, economic evaluation of various treatment options is complex and palliative chemotherapy particularly should not just be dismissed as inappropriately expensive; the costs of the drugs used and the in-patient, out-patient, and home care required must all be assessed, and compared with those of 'best supportive care' where, for example, more time may be spent by the patient in hospital than when having active treatment (Jaakkimainen *et al.*, 1990). Also, in non-specialist hands the approach to palliative anti-cancer treatment may be very nihilistic; 'more' may not be better but 'less' is sometimes inadequate. For example, continuous chemotherapy is better than intermittent chemotherapy for advanced breast cancer (Coates *et al.*, 1987). Dedicated oncologists should be available to advise in such situations and consensus guidelines for the management of common cancers established by expert panels (Timothy, 1988).

Patients and their relatives have different perspectives than their professional carers. Undoubtedly patients with cancer are more likely to opt for radical treatment with minimal chance of benefit, than people who do not have cancer, including medical and nursing professionals (Slevin *et al.*, 1990). If palliation is the accepted aim, good quality of life becomes the top priority. However, in many clinical trials this has not been assessed and it is acknowledged that whereas quality of life is rarely improved by treatment that does not lead to tumour response, the latter may not correlate well with patient well-being. Quality of life as judged by patients themselves is different from that as assessed by their doctors and nurses (Slevin *et al.*, 1988). Nevertheless, physical function, mood, physical symptoms and social support are the key elements in assessment, and these should be monitored from diagnosis, through treatment and in 'terminal' illness (Mor, 1987). In the latter phase the 'process' of care rather than the 'outcome' must be assessed and this is not straight-forward since care is more than usually multidisciplinary and specific measures to focus on the perception of the relatives rather than the person who is dying are also needed (NHS Management Executive, 1991). Quality must be the endpoint however (Ahmedzai, 1990) and this should be assessed against the background of cost effectiveness and limited resources. Palliative care can be undertaken in hospital (including specialist cancer treatment centres), in hospices or at home. Most people would prefer to die at home (Dunlop *et al.*, 1989) with a hospice as second choice; despite this about three quarters die in hospital, and proportionately few die in a hospice. Superficially, home care would appear to be a cheap option for the NHS but if the increased work load of the community team, the higher cost of community prescribing, the specialist care services required and the extra care and support needed for the patient's family are taken into account home care costs can be as high as those in institutions (Gray *et al.*, 1987). Hospice care represents excellent value to the NHS which provides overall less than half of the funding of hospices. Bed costs in hospices vary according to size but are probably about the same as those of an acute general hospital (Hill & Oliver, 1988, Dunt *et al.*, 1989), which currently average about £110 per day (with the caveat that there are many variables in different settings; for example, this figure is likely to be higher in larger and

Received 30 August 1991; and in revised form 24 September 1991.

specialist institutions). If in fact there is little to choose cost-wise between hospital, home and hospice, then in the ideal situation the choice of where to die should be made by the patients themselves, though inevitably any clinical decision will be affected by what is locally available.

Much work still needs to be done on consensus management, audit and quality assurance (Standing Medical Advisory Committee, 1990) in the field of palliation. Initiatives such as The Trent Region Palliative Care Centre should serve as development foci for all facets of the management of incurable disease and lead and stimulate effective research and teaching in this multidisciplinary field.

Oncologists and palliative care physicians, or hybrids of the two (McIllMurrary, 1987), should lead the way and work together in initiating the new NHS changes to the future benefit of our patients (Hancock, in press).

### References

AHMEDZAI, S. (1990). Palliative care in oncology: making quality the end point. *Ann. Oncol.*, **1**, 396.

COATES, A., GEBSKI, V., BISHOP, J.F. & 12 others (1987). Improving the quality of life during chemotherapy for advanced breast cancer. *New Eng. J. Med.*, **317**, 1490.

DUNLOP, R.J., DAVIES, R.J. & HOCKLEY, J.M. (1989). Preferred versus actual place of death: a hospital palliative care support team experience. *Pall. Med.*, **3**, 197.

DUNT, D.R., CANTWELL, A.M. & TEMPLE-SMITH, M.J. (1989). The cost effectiveness of the City Mission Hospice programme, Melbourne. *Pall. Med.*, **3**, 125.

GRAY, D., MACADAM, D. & BOLDY, D. (1987). Comparative cost analysis of terminal cancer care in home, hospice patients and controls. *J. Chron. Dis.*, **40**, 801.

HANCOCK, B.W. (1991). Future directions for the clinical management of cancer. In *Medicine and management: cancer – the next decade. Proceedings of the tenth Trent Regional Health Authority Seminar 1990.* Pemberton, J. (ed.) (in press), Trent RHA.

HILL, F. & OLIVER, C. (1988). Hospice – an update on the cost of patient care. *Health Trends*, **20**, 83.

JAAKKIMAINEN, L., GOODWIN, P.J., PATER, J., WARDE, P., MURRAY, N. & RAPP, E. (1990). Counting the costs of chemotherapy in a National Cancer Institute of Canada randomized trial in the non small-cell lung cancer. *J. Clin. Oncol.*, **8**, 1301.

MARKMAN, M. (1988). An argument in support of cost-effectiveness analysis of oncology. *J. Clin. Oncol.*, **6**, 937.

MCILLMURRAY, M.B. (1987). District cancer physicians: report of a working group of the Association of Cancer Physicians. *J. Roy. Coll. Phys.*, **21**, 117.

MOR, V. (1987). Cancer patient's quality of life over the disease course: lessons from the real world. *J. Chron. Dis.*, **40**, 535.

MRC LUNG CANCER WORKING PARTY (1991). Inoperable non-small-cell lung cancer: a Medical Research Council randomized trial of palliative radiotherapy with two fractions or ten fractions. *Br. J. Cancer*, **63**, 245.

N.H.S. MANAGEMENT EXECUTIVE (1991). Funding of hospices and similar organizations: guidelines for developing contracts for services for the dying and bereaved. EL (91) 38 annex B.

PRICE, P., HOSKIN, P.J., EASTON, D., AUSTIN, D., PALMER, S.G. & YARNOLD, J.R. (1986). Prospective randomised trial of single and multifraction radiotherapy schedules in the treatment of painful boney metastases. *Radiother. Oncol.*, **6**, 247.

REES, G.J.G. (1985). Cost effectiveness in oncology. *Lancet*, **ii**, 1405.

REES, G.J.G. (1991). Cancer: deciding what we can afford. *Br. Med. J.*, **302**, 799.

REES, G.J.G., DEUTSCH, G.P., DUNLOP, P.C.R. & PRIESTMAN, T.J. (1991). Clinical oncology services to district general hospitals: report of working party of the Royal College of Radiologists. *Clin. Oncol.*, **3**, 41.

RUBENS, R.D. (1990). Auditing palliative cancer chemotherapy. *Eur. J. Cancer*, **26**, 1023.

SLEVIN, M.L., PLANT, H., LYNCH, D., DRINKWATER, J. & GREGORY, W.M. (1988). Who should measure the quality of life, the doctor or the patient. *Br. J. Cancer*, **57**, 109.

SLEVIN, M.L., STUBBS, L., PLANT, H.J. & 4 others (1990). Attitudes to chemotherapy: comparing views of patients with cancer with those of doctors, nurses and general public. *Br. Med. J.*, **300**, 1458.

SPIRO, S.G. & SOUHAMI, R.L. (1990). Duration of chemotherapy in small cell lung cancer. *Thorax*, **45**, 1.

STANDING MEDICAL ADVISORY COMMITTEE DEPARTMENT OF HEALTH. (1990). *The Quality of Medical Care*. London: HMSO.

TIMOTHY, A.R. (1988). Cost versus benefit in non-surgical management of patients with cancer. *Br. Med. J.*, **297**, 471.